# Pathological Fracture as the Initial Presentation of Metastatic Pancreatic Cancer

**DOI:** 10.7759/cureus.20920

**Published:** 2022-01-04

**Authors:** Dunya Alfaraj, Rawan O AlOtaibi, Reema M Bamousa, Jana F Alzamami

**Affiliations:** 1 Emergency Department, Imam Abdulrahman Bin Faisal University, King Fahad University Hospital, Dammam, SAU; 2 Medicine, Imam Abdulrahman Bin Faisal University, Dammam, SAU

**Keywords:** pancreatic adenocarcinoma, pancreatic cancer, spinal fracture, pathological fractures, skeletal metastasis

## Abstract

Pancreatic cancer with bone metastasis is considered a very rare malignancy. Although the incidence rate of people with pancreatic cancer who develop bone metastasis is unknown, it is estimated as between 5% and 20%. We report a 61-year-old lady who had multiple emergency department visits complaining of lower back pain radiating to the lower limbs for five months. Clinical and imaging workup suggested pancreatic adenocarcinoma metastasis to the second lumbar vertebra (L2), lungs, and ovaries.

## Introduction

Pancreatic cancer accounts for about 3% of all cancer in the United States and is about 7% of the cause of mortality among cancers worldwide, with a lifetime risk of about 1 in 64 [1]. The most common sites of metastases in pancreatic cancer are the liver and peritoneum. Other less common sites include the lung, brain, kidney, and bone [2-3]. Although the prevalence rate of skeletal metastases in patients with pancreatic cancer is unknown, it is estimated to be between 5% and 20% [3]. According to the American Cancer Society, the five-year relative survival rate in metastatic pancreatic cancer patients is about 3% [4]. In Italy, a case of pancreatic cancer was reported with vertebral fracture and spinal cord compression [5]. In China, they reported a case of pancreatic cancer with a vertebral compression fracture [6]. In Athens, vertebral metastasis was found in a case of pancreas cancer with back pain as an initial presentation [3]. In another case reported in Japan, they found right iliac bone and spinal metastases in a patient with pancreatic cancer [7]. Furthermore, in the USA, they reported a case of pancreatic cancer with right clavicular head metastases [8]. One more case in the USA reported sacrum metastasis as the only site in a patient seven years after treatment for a locally advanced pancreatic adenocarcinoma [9].

## Case presentation

**Figure 1:**
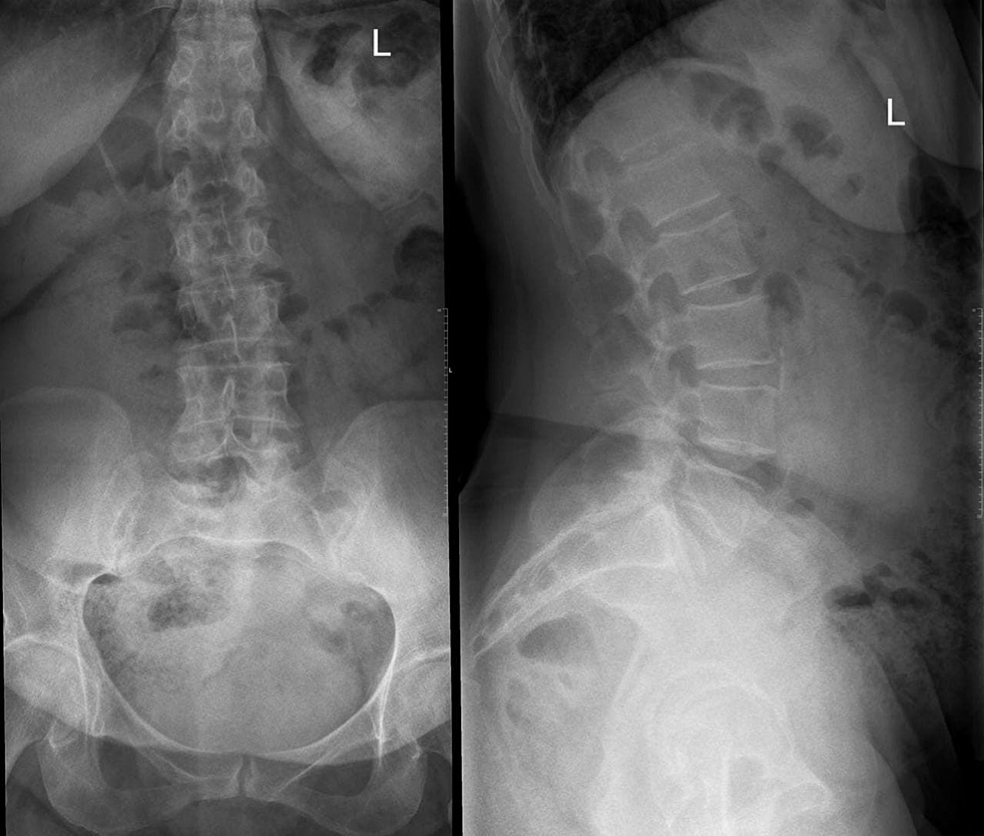
X-ray of the lumbar spine; anterior-posterior (AP) and lateral views Showing unremarkable lumbar spine

**Figure 2:**
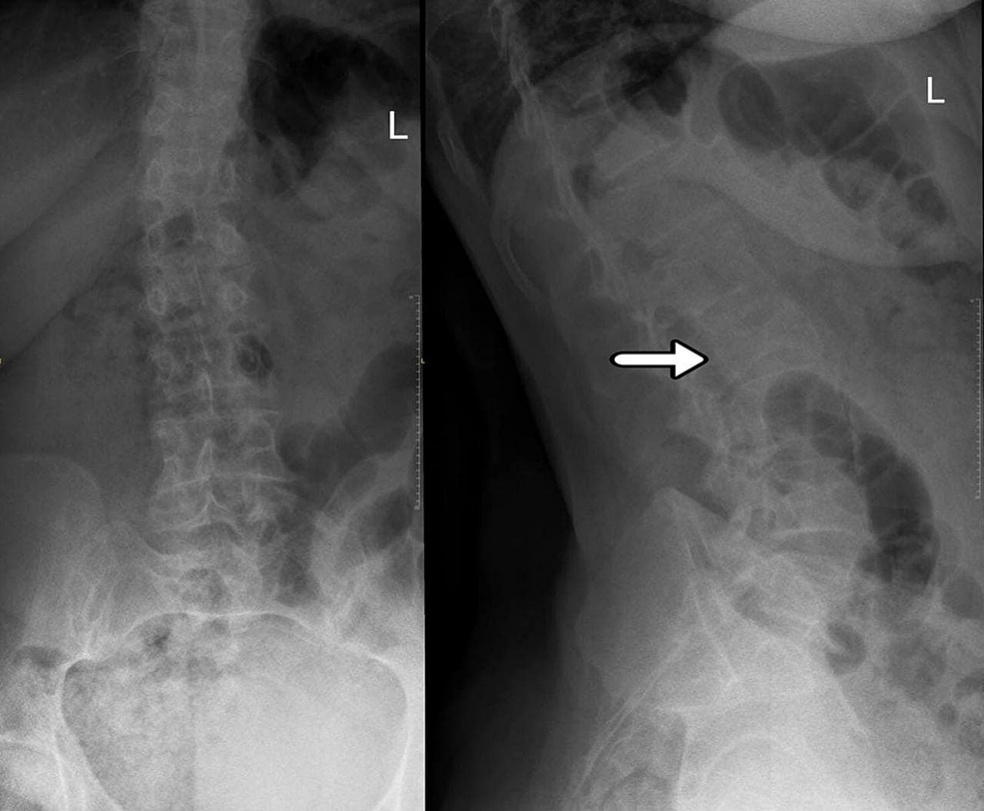
X-ray of the lumbar spine; AP and lateral views Showing L2 compression, with no obvious fracture

**Figure 3:**
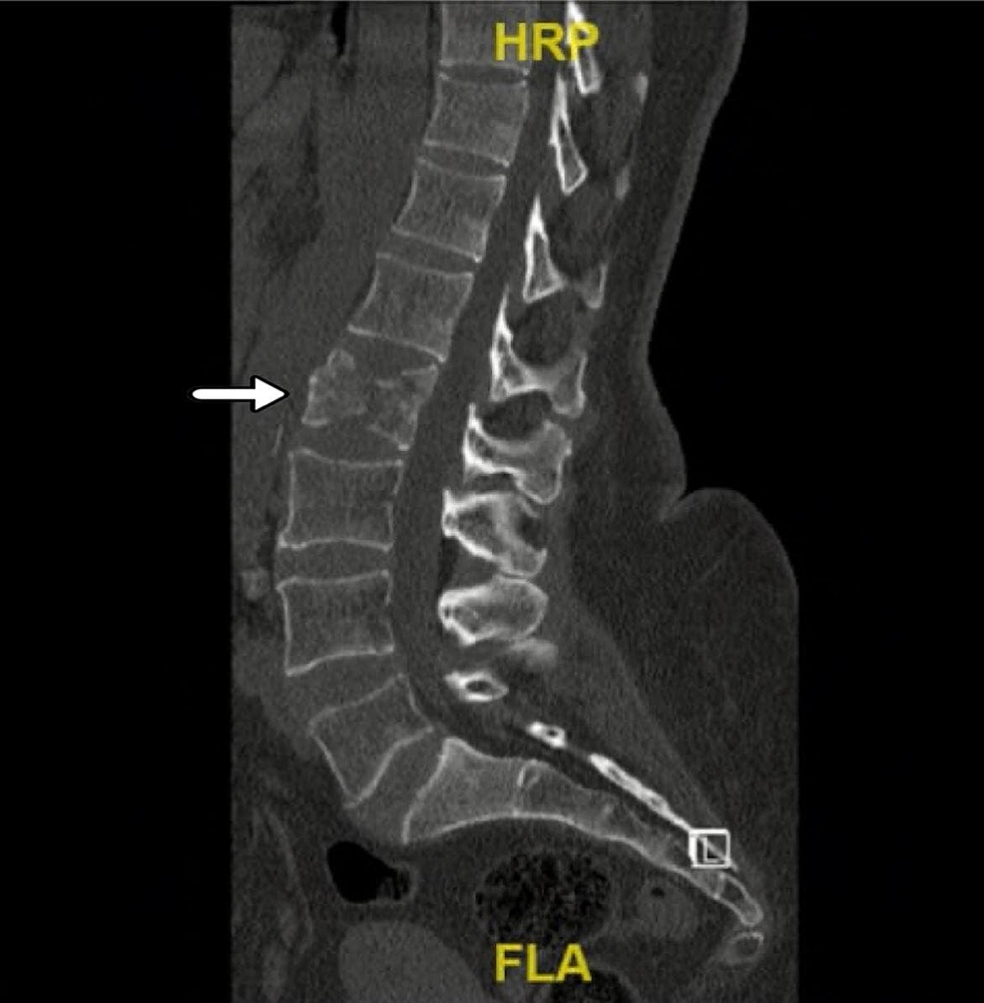
CT scan lumbar without contrast Showing L2 vertebral body compression fracture with no definitive compression on the spinal cord

Initially, a 61-year-old Saudi female patient with a known case of controlled hypertension came to the emergency department (ED) complaining of low back pain that started one day ago after she slept; however, she hadn't suffered a fall. The pain was exacerbated by movement, not radiating to the lower limbs, and not associated with any neurological defects. Physical and neurological examinations were unremarkable. In addition, an X-ray of the lumbar spine was done (Figure 1) and no findings were reported so the patient was discharged on conservative management. A few weeks later, she had several visits to the ED with the same complaint, and her pain was increasing, which made her unable to sit or lie supine for long periods. A decreased range of motion of the spine was noticed, but no numbness or weakness was reported. The general physical examination was normal, and the lower limb neurological examination showed bilateral hip flexion power of four out of five at the L2 level, positive right side Babinski sign, and bilateral intact sensation at all levels. During the same visit, an X-ray of the lumbar spine was done (Figure 2) as well as a CT of the lumbar spine, which showed an L2 vertebral body compression fracture with no definite compression on the spinal cord or retropulsion fragment and normal CT features of the paravertebral soft tissues (Figure 3). However, since the patient’s condition improved, she was discharged from the ED and was referred to the orthopedic spine clinic for follow-up.

**Figure 4:**
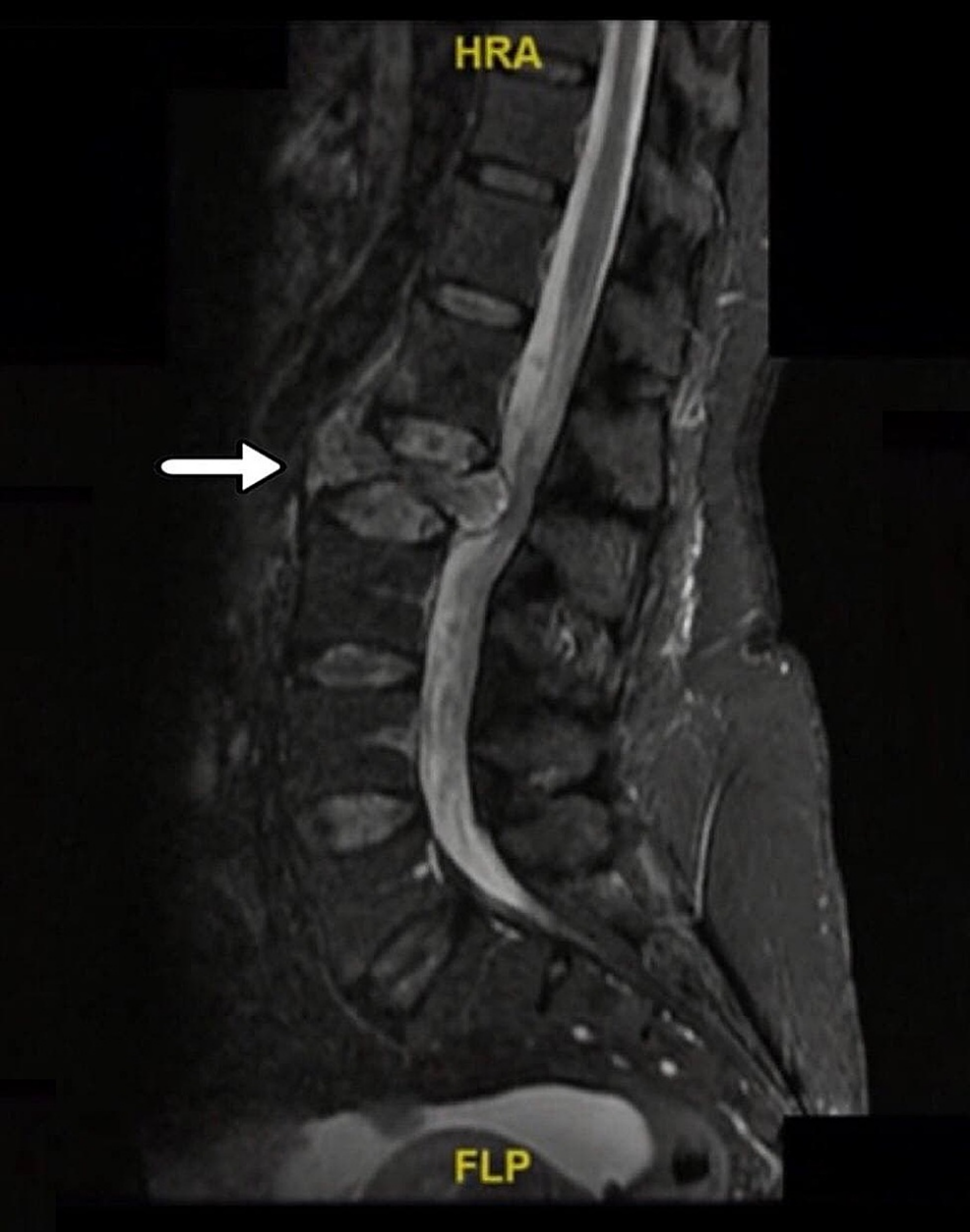
MRI scan lumbar spine Showing L2 compression fracture with significant height reduction

The patient was following up regularly with the orthopedic spine clinic and had multiple ED visits before admission. However, three months after her first ED visit, the patient was admitted through the outpatient clinic for further investigations since her back pain was progressively increasing with no improvement. During the admission, a lumbar spine MRI (Figure 4) was done, which concluded an interval progression of L2 compression fracture with significant height reduction compared to the old CT that was done three months prior to her admission. Moreover, a paravertebral component and retropulsion were noticed that were compressing the cauda equina causing severe spinal canal stenosis and bilateral neural foraminal narrowing.

She also underwent chest abdomen pelvis CT (CAP CT) scan, and multiple scattered pulmonary nodules were found as well as a poorly defined pancreatic tail hypodense mass measuring 3.9 cm, bilateral complex cystic ovarian lesions were found most likely metastatic lesions, and overly distended gallbladder containing gallstones with no evidence of cholecystitis.

The patient was scheduled for posterior instrumented spinal fusion to regain spine stability and open pedicular biopsy from the vertebral body (L2). The gross histopathological description showed multiple tan/brown firm to hard tissue fragments measuring in aggregate 1.4 X 1.2 X 0.2 cm and the immunochemical panel results came out as the following: CK7 and CK19: Positive; CK20, PAX8, and TTF1: Negative. Moreover, the radiologic finding with the elevated tumor marker (CA 19-9) along with the histopathological and immunochemical profile are in favor of primary pancreatic origin.

The patient and her family were informed and educated about the diagnosis, prognosis, and treatment options on multiple sessions, and the patient was transferred to a tertiary hospital for further evaluation, management, and follow-up.

## Discussion

Pancreatic cancer is considered the seventh cause of cancer-related deaths worldwide. It is described as an aggressive type of cancer that has a very poor prognosis and high mortality rates [1-10]. According to Torre et al. (2012), the incidence of pancreatic cancer in developed countries and less developed countries was found to be 1% and 0.4%, respectively [10]. In addition, the five-year survival rate was found to be less than 5% in patients with pancreatic cancer [4-11]. The most common sites of pancreatic cancer metastasis are the liver and peritoneum. However, the brain, lungs, kidneys, and bones are considered unusual sites for metastasis [2-3]. According to Borad et al. (2009), the spine was the most common site of skeletal metastasis in the case of an advanced stage of pancreatic cancer. As back pain might be a symptom of pancreatic cancer itself, but it could be an indication of bone metastasis as well [2]. Usually, in the early stages of pancreatic cancer, patients would be asymptomatic. However, the diagnosis mostly depends on the symptoms, which indicates late stages and, thus, poor prognosis. In some cases, the pancreatic tumor is suspected due to a predisposing condition, which will lead to an early diagnosis and a better prognosis. The symptoms of pancreatic cancer include abdominal pain, anorexia, early satiety, insomnia, and weight loss. Moreover, back pain might be present, and it would indicate an unresectable tumor, poor prognosis, and low survival rates [12]. In patients with suspicion of pancreatic cancer, laboratory investigations, including complete blood count (CBC), liver enzymes to exclude biliary obstruction, fasting glucose, and glycated hemoglobin (HBA1C) to rule out diabetes. Patients with jaundice or nonspecific abdominal pain can initially undergo an ultrasound followed by CT if pancreatic cancer is suspected. Patients with contraindications of CT would undergo MRI. If imaging results are unclear, endoscopic ultrasound- or CT-guided biopsies can be used for further evaluations [13]. According to Puri et al. (2021), skeletal metastases in pancreatic cancer are considered uncommon, a sample included 207 patients who were diagnosed with advanced pancreatic cancer, during the evaluation of these patients, only 19 (9.2%) were detected with skeletal metastasis. The thoracic and lumbar spine was the most common site for skeletal metastasis. The initial observed symptom was bone pain in seven out of 19, pathological fractures were two out of 19, and one out of 19 patients had inferior vena cava compression secondary to para-spinal mass [14]. In our case, the patient presented with low back pain and decreased range of motion due to compression fracture of the spine, the patient underwent lumbar spine CT, which showed L2 compression due to metastasis. After further workup patient was diagnosed incidentally with pathological metastatic adenocarcinoma. Surgical resection of the tumor is considered the only curative management for pancreatic cancer. However, most of the patients will present with locally advanced unresectable lesions. Some studies suggested the use of chemoradiation therapy to convert the unresectable lesions to surgically resectable lesions, which eventually will have the same prognosis as patients who were diagnosed initially with resectable lesions, but this method is still controversial [15]. In case of locally advanced lesions with metastasis, the National Comprehensive Cancer Network recommends starting with FOLFIRINOX or gemcitabine monotherapy to relieve the symptoms and slightly improve the survival rate (several months). Moreover, a combination of gemcitabine and erlotinib, capecitabine, or oxaliplatin can be used as the second-line therapy for advanced stages of pancreatic cancer [15-16]. However, radiation therapy is only indicated in patients who progress metastatic disease and are in need of palliative care [17]. Survival periods of pancreatic cancer patients with bone metastases are usually not long, however, treatment for bone metastases is important in terms of quality of life. An earlier diagnosis is essential to prevent deterioration in the quality of life of pancreatic cancer patients presenting with bone metastases [18]. In our case, our patient underwent an open pedicular biopsy from the vertebral body (L2). The pathology report confirmed the metastasis, the patient was informed about the pathology report and referred to a tertiary hospital for a further management plan.

## Conclusions

There are limited examples of pathological fractures as the initial presentation of metastatic pancreatobiliary adenocarcinoma in the literature. This case illustrates the unusual dissemination of pancreatobiliary adenocarcinoma. However, elderly patients with bone pain and pathological fractures must be taken seriously and a full investigation must be done to rule out bone metastasis and malignancy before they reach an advanced stage. In conclusion, considering back pain and pathological fractures as bone metastasis will help in reaching the appropriate diagnosis and will give the patient appropriate care and, hopefully, a better prognosis.
